# Peri-Operative Care of Technology-Dependent Adolescents and Young Adults

**DOI:** 10.3390/children12040417

**Published:** 2025-03-26

**Authors:** Jia Liu, Robert J. Graham, Shawn S. Jackson

**Affiliations:** 1 Department of Anesthesiology, Pain and Perioperative Medicine, Children’s National Hospital, Washington, DC 20010, USA; 2 Department of Critical Care Medicine, Children’s National Hospital, Washington, DC 20010, USA; 3 Department of Anesthesiology, Critical Care, and Pain Medicine, Boston Children’s Hospital, Boston, MA 02115, USA; robert.graham@childrens.harvard.edu (R.J.G.);

**Keywords:** medical complexity, technology dependence, tracheostomy, pediatric anesthesia, peri-operative optimization, guardianship

## Abstract

Caring for technology-dependent adolescents and young adults presents significant challenges, especially for procedural and peri-operative care. This review delves into the complexities of managing these patients before, during, and after major medical procedures or operations, highlighting the unique medical and psychosocial issues that demand specialized attention. We address the intricacies of pre-procedural assessment and optimization, as well as post-procedural management, with a particular focus on the challenges associated with life-sustaining technologies such as chronic ventilator dependence. Additionally, we explore medicolegal factors such as guardianship and surrogate decision-making, which are often more complex in this population. The review also identifies key areas of uncertainty that merit further research and exploration, aiming to enhance the quality of care and improve outcomes for technology-dependent individuals transitioning to adult healthcare.

## 1. Introduction

The population of technology-dependent children in the United States has grown dramatically and represents a substantial number of pediatric outpatient visits and hospitalizations [1]. This group of patients includes those who require supplemental oxygen, non-invasive and invasive ventilation [2,3], parenteral nutrition, or enteral nutrition through a device such as a gastrostomy or gastrojejunostomy tube [4]. As the survival of technology-dependent pediatric patients continues to improve, the number of technology-dependent adolescents and young adults (AYAs) continues to increase [5,6]. The challenges associated with transitions of care from pediatric to adult healthcare systems, particularly in adolescence, have been well described in the population at large [7,8], but transitions in the technology-dependent cohort are less well characterized. These patients face a multitude of challenges beyond the ordinary scope of medical care, influenced by both clinical issues and psychosocial dynamics.

The recent consolidation of pediatric care highlights the limited pediatric resources currently available [9,10] and is one of the drivers of transitions for AYAs. Independent of the patient and family experience, which is often the driver to remain at a pediatric facility, there is a “medical” tipping point where screening and management are more effectively addressed within adult programs. As this population with complex medical needs ages, they also develop age-related conditions with which pediatric providers are less familiar or less up to date. Pregnancy in a young woman with SMA is a clear demarcation, but most require thoughtful and gradual introduction/integration. Conversely, the care of young adults with complex congenital conditions (e.g., cyanotic heart lesions), as well as significant cumulative morbidities, requires adult providers to reacquaint themselves with these issues and adapt multidisciplinary service lines to best address the care coordination needs.

The peri-operative or peri-procedural setting amplifies these challenges, presenting unique circumstances that demand seamless integration of technological support and comprehensive care strategies [11]. Technology-dependent AYAs may require major medical procedures or surgeries as they are transitioning between pediatric and adult healthcare systems. These can include device placement or maintenance (e.g., gastrostomy or tracheostomy), procedures to optimize function or mitigate cumulative morbidity (e.g., spinal fusions or pelvic osteotomies), procedures related to technology failure (e.g., ventriculoperitoneal shunt revisions, tracheostomy site revisions), or unrelated surgical issues. Routine screening procedures for adult patients, such as endoscopy and colonoscopy, may also be required. Adults admitted to pediatric intensive care units (PICUs) often have complex chronic conditions and greater severity of illness when compared with younger patients admitted to the PICU. A majority of these admissions are often elective and peri-operative, with a higher proportion of older corresponding ages [12]. The complexity and underlying diagnoses of the patient and available surgical expertise should guide healthcare providers when deciding whether the procedure and subsequent recovery should take place in pediatric or adult centers. Critical to this process is the involvement of a multidisciplinary team, bridging pediatric and adult care while encompassing surgery, anesthesiology, primary care, subspecialty services, social work, and ethics. Together, they must navigate the intricacies of pre-operative optimization and post-rehabilitation while ensuring continuity in the management of life-sustaining technologies. Additionally, medicolegal considerations, such as the nuances of guardianship, limited or extended, and surrogate decision-making, add a layer of complexity that requires specialized understanding and sensitivity.

Despite advancements in medical technology and care protocols, significant gaps persist in our knowledge and practice related to this population [13]. This review aims to identify these areas of uncertainty, advocating for a focused research agenda that addresses the specific needs of technology-dependent AYAs making the transition to adult healthcare services. By shedding light on these issues, we aspire to contribute to a more integrated and effective approach to peri-operative care for this vulnerable and growing population.

## 2. Methods

A comprehensive literature search was conducted using the PubMed database from 1 January 2005 to 1 January 2025 for this narrative review. Articles pertaining to peri-operative care of children and adults with medical complexity and technology dependence were searched. Article selection was performed at the discretion of the authors and included abstracts, clinical and narrative reviews, case series, and retrospective and prospective cohort studies. Given the paucity of published data on this topic, the inclusion of expert opinions from the authors, who have significant clinical and academic experience with this patient population, was necessary to provide a comprehensive review.

## 3. Medical Decision-Making and Guardianship

The transition from pediatric to adult healthcare introduces complex consent and medicolegal challenges for technology-dependent patients. These individuals often reach the age of legal competence—typically 18—while still under significant medical oversight, necessitating a careful reevaluation of their legal status and decision-making capacities. This transition represents a major change in practice for most patients, as pediatric patients under the age of 18 are usually only involved in assent, but not necessarily in consent (outside of unique situations). As such, while the patient is a minor, they have a surrogate medical decision-maker, most frequently their parents, who make consent decisions on their behalf [14,15]. Even more importantly, parental consent is required irrespective of the patient’s cognitive ability or degree of technological dependence, and no legal paperwork is required when the parents are acting on behalf of their child. However, many of these issues become central to the discussion upon the transition to the age of majority. The framework by which these issues can be addressed is as follows.

### 3.1. Legal Transition to Adulthood

As patients turn 18, they are legally recognized as adults and are granted the autonomy to make their own medical decisions. This legal transition requires healthcare providers to reassess consent processes and to ensure patients understand their rights and responsibilities. Education and support are vital to empower these young adults to participate actively in their care decisions, when appropriate.

### 3.2. Guardianship and Surrogate Decision-Making

Some technology-dependent AYAs may have cognitive or developmental impairments that limit their capacity to make informed decisions. In such cases, guardianship or surrogate decision-makers must be legally appointed once the patients are over the age of 18. Identifying and confirming legal guardianship ensures that decisions are made in the AYA’s best interest, adhering to both ethical standards and legal requirements. Guardianship requirements and processes are unique to the Country (and State) in which the patient resides and are subject to nuanced interpretations of local laws. Notably, in many jurisdictions, establishing guardianship can be a long process. Thus, preparations for guardianship should begin in the patient’s mid-teens with the intent to file in the year before the patient turns 18 to avoid disruptions in care as these patients transition into legal adulthood [16]. If major medical procedures are being considered for a patient who is approaching legal adulthood, guardianship or a surrogate decision-maker should be established prior to proceeding with the procedure, as these patients are at higher risk for prolonged hospital stays and could be left hospitalized without a legal decision-maker.

### 3.3. Advance Directives and Transition Planning

Advance directives have become an important consideration for technology-dependent patients transitioning to adult care. Developing these documents can help articulate the patient’s preferences for care, especially in peri-operative and end-of-life situations. Encouraging discussions about advance care planning as part of the transition process can provide clarity and peace of mind for both patients and families. The peri-operative period can also be an opportunity to discuss the need to create or amend existing advance directives.

### 3.4. Confidentiality and Privacy

With adulthood comes the renewed responsibility to protect patient confidentiality and privacy. Healthcare providers must navigate the balance between respecting patient autonomy and involving family members who may have played a crucial role in care up to this point. Clear, patient-focused communication and consent discussions can help mitigate potential conflicts and ensure all parties are informed and aligned.

### 3.5. Ethical Considerations

The shift in decision-making power can raise ethical dilemmas, particularly when there are discrepancies between a patient’s wishes and those of their family or caregivers around invasive medical procedures. In most cases, patient autonomy is often paramount. However, engagement with primary care and multidisciplinary ethics consultations, particularly in hospitals that have provided longitudinal care for the patient, can be valuable in resolving conflicts and ensuring that care plans align with ethical principles and the patient’s values and preferences.

Addressing these consent and medicolegal issues is critical to providing comprehensive, respectful, and patient-centered care. By aligning legal considerations with clinical practices, healthcare providers can support a smoother transition and ensure that technology-dependent adolescents moving into adult care are afforded the dignity and autonomy they deserve.

## 4. Pre-Operative and Pre-Procedural Planning

Preparation for these patients is best achieved well in advance of the procedure or operation in question and take into consideration the topics provided in Table 1. While satisfying the medicolegal issues above is a necessary pre-requisite, it is not sufficient in terms of optimization and planning. With medically complex and technology-dependent patients, optimization often involves input from a variety of surgical and non-surgical specialties. Preoperative interventions can be focused on optimizing specific organ functions, such as nutrition and hematology. A broader approach utilizes preoperative visits and multidisciplinary interventions, incorporating hospitalists, complex care clinicians, and others to optimize preoperative health and safety, often conducted weeks to months before anticipated surgery [17]. In fact, the peri-operative home model frequently demonstrates its value most abundantly with these patients and may also serve as an opportunity to begin discussions around the transition to adult care [18,19]. For patients who have already transitioned to adult care, adult providers should be encouraged to involve pediatric subspecialty colleagues and reference the pediatric literature [20,21,22].

Preoperative risk assessment should be a routine part of the preoperative evaluation for these patients, as comorbidities and end-organ dysfunction often accumulate as these patients grow into adulthood. ASA status is often not an adequate risk stratification for these patients, and alternative preoperative risk stratification in this complex patient population should be considered. While not widely adopted, the NARCO-SS has been shown to perform better than ASA physical status for children with neuromuscular diseases and may warrant inclusion when conducting the preoperative evaluation for these patients [23,24].

## 5. Intra-Operative and Peri-Procedural Management

Management of the patient while in the hospital for a procedure or operation frequently requires the deployment of a skilled and multi-disciplinary team.

Importantly, there exist numerous anesthetic considerations for these high-complexity patients as noted in Table 2. This management can be particularly critical, as the choice of anesthetic technique may be dependent on both pre-operative factors as well as the goals for hospitalization. Ideally, the anesthesia team that will be managing the patient on the day of the procedure should be identified in advance so they can work with the surgical and procedural teams to help with optimization and ensure smooth execution of the anesthetic plan on the day of the procedure [25].

Other important considerations include nursing team involvement (particularly with multi-disciplinary interventions) and the coordination of other hospital resources, such as the ICU (if indicated), laboratory medicine, pre-op, and PACU.

## 6. Post-Operative Care and Follow-Up

Post-operative care for technology-dependent patients requires meticulous planning and seamless coordination to ensure optimal recovery and continued health maintenance. While no data specific to AYAs with technology dependence exists, children with technology dependence have been shown to have a 3-fold longer hospital length of stay, a 4-fold increased risk of mortality, and more frequent readmissions compared to other children with medical complexity [28,29,30]. This phase of care is critical, as it involves addressing both the immediate aftermath of surgery and the long-term support needs of the patient, who often relies on complex medical devices.

The immediate post-operative period is characterized by initial recovery from the procedure and anesthetic. This may be completed in the PACU or the ICU setting depending on institutional and patient factors. Care teams must pay particular attention to the functionality and integration of life-sustaining technologies, such as ventilators, feeding tubes, enteral/parenteral nutrition advancement, resumption of routine medication regimens, or dialysis machines. Aggressive airway clearance for patients with neuromuscular disease is often necessary in addition to their baseline respiratory support. Bowel motility may be adversely affected due to anesthetic/pain medications or because of the surgery itself, and early initiation of a bowel regimen or prokinetic agent should be considered. Adjustments may be required to accommodate changes brought about by the surgical procedure, necessitating close collaboration among surgeons, anesthesiologists, and technology specialists.

Patients requiring hospital admission should undergo a thorough medication reconciliation on admission and prior to discharge, as medication errors are more prevalent in this patient population [31]. Vital home medications, such as antiepileptics and dystonia medications, may require a parenteral transition of home medications or conversion to parenteral alternatives following surgery until enteral feeds are resumed.

Tailored pain management strategies are essential, taking into account the patient’s unique physiological and psychological needs. Adequate pain control is vital for ensuring adequate ventilation after surgery; however, titrations should be made with care in this population. Care should be taken in this population to decrease the risk of iatrogenic injuries, such as fractures and pressure injuries. Additionally, early initiation of rehabilitation and physical therapy can aid in a smoother recovery process, promoting mobility and preventing deconditioning.

Effective long-term follow-up involves regular assessments to monitor recovery progress and the ongoing functionality of medical devices. Transitioning from pediatric to adult care often necessitates continued collaboration between the two realms, ensuring comprehensive support. This includes setting up appropriate referrals, providing education on self-management skills, and supporting adherence to follow-up appointments and treatment plans.

## 7. Limitations

As a narrative review, there are limitations inherent to the type of review that was conducted. While a thorough literature review search was conducted, there is still inherent bias as the search was not conducted in a systematic way and is, therefore, at risk of error. Due to the paucity of existing data, this review also relied on expert opinion, which can have its own biases as well.

## 8. Conclusions

The transition from pediatric to adult healthcare services for technology-dependent patients represents a unique juncture that requires thorough attention and planning, particularly in the peri-operative setting. As these young individuals and their families navigate this transition, they encounter a host of challenges, including shifts in medical responsibility, psychosocial dynamics, and medicolegal status. Effective peri-operative management is crucial to ensuring that their complex needs are met with competence and compassion.

By integrating strategies for pre-operative optimization, intra-operative management, and post-operative follow-up, healthcare providers can foster continuity and improve outcomes. Key areas for improvement include enhancing communication among stakeholders, promoting patient education, and addressing gaps in research that can inform better practices.

Looking ahead, it is essential that healthcare systems strengthen the frameworks facilitating these transitions, ensuring they are equipped to handle the multifaceted needs of technology-dependent patients, whether through primary care physicians or the use of a peri-operative surgical/medical home model. By prioritizing patient-centered approaches and engaging in continuous research and education, we can support these young individuals in achieving optimal health and quality of life as they move into adulthood.

Ultimately, the commitment to understanding and addressing the comprehensive needs of this population not only enhances the care experience but also paves the way for future innovations and advancements in transitional healthcare.

## Figures and Tables

**Table 1:** Perioperative Considerations for Preoperative Planning.

Pre-Operative Component	Description
Identification of primary care physician or medical/surgical home.	In the peri-procedural period, it is helpful to have a centralized physician (either primary care or via a medical/surgical home model). This provider would be responsible for coordinating with subspecialists to ensure that the patient is medically optimized prior to the scheduled procedure.
Ensuring sub-specialty commitment	For technology-dependent patients, care is often provided by numerous subspecialty services. If these services are expected to be required as consultants during an upcoming procedure, this should be confirmed prior to the procedure in question and preoperative recommendations should be elicited. Preparations may be required to transition care at the subspecialty level via clinic visits prior to the procedure. When possible, avoid fragmentation of care between pediatric and adult providers/institutions when possible.
Preoperative Optimization	Thorough preoperative evaluation and optimization can often be accomplished as an outpatient in the presence of an established medical/surgical home. Initiation of a pulmonary sick plan or escalation of baseline pulmonary clearance prior to surgery should be considered. Optimization of preoperative electrolytes and blood counts may be necessary. Patients with limited fasting capabilities may require preoperative admission for initiation of preoperative intravenous fluids. Low threshold to defer anesthesia and non-emergent procedures during intercurrent illness.
Contingency Planning	Consider peri-operative contingency planning and ensure expertise is available at the center where the procedure is to take place. This includes a discussion of the need for post-operative admission to the intensive care unit.
Rehabilitation Goals (if necessary)	Identify clear rehabilitation goals and determine if these goals can be achieved via discharge to home or whether additional inpatient rehabilitation may be necessary.
Discharge Criteria	Identify discharge criteria and any barriers to post-operative care with input from continuity subspecialty providers. For instance, special considerations and staffing may need to be made for ventilator-dependent patients to recover in the PACU for a day surgery procedure. Patients who are fed via enteral tubes will require the appropriate tubing and syringes to advance their diet safely prior to discharge. Challenges associated with disruption of home care services around the peri-operative period should be taken into consideration, as lack of adequate home nursing or appropriate transportation could delay discharge from the hospital despite medical readiness.

**Table 2:** Anesthetic Considerations.

Anesthetic Considerations	
Airway Management	Craniofacial abnormalities or impaired head/neck mobility can contribute to difficulty with mask ventilation and/or intubation. Preoperative airway assessment and review of prior anesthetics are important in ensuring adequate preparation in the event of a difficult airway. Decision-making around the timing of extubation should take into consideration any difficulty with intubation, baseline respiratory function and support, and the complexity of the surgery.
Anesthetic Technique and Medications	It can be essential to minimize GA or opiates in patients with a tenuous respiratory status at baseline. When possible, a primary regional or neuraxial technique should be considered. Feasibility may play a role in decision-making as many patients have associated comorbidities that may limit the ability for regional anesthetic techniques to be successful, such as scoliosis or prior orthopedic surgery. Succinylcholine is contraindicated in patients with certain muscular dystrophies, such as Duchenne muscular dystrophy and Becker muscular dystrophy, due to the risk of rhabdomyolysis and/or severe hyperkalemia [26]. When neuromuscular blockade is required, careful monitoring of neuromuscular function via train-of-four testing should be used throughout, as the safety and efficacy of reversal agents such as sugammadex have yet to be validated in patients with neuromuscular disease, although they have been increasingly adopted [27]. Triggering agents such as succinylcholine and inhaled anesthetics should be avoided in patients with underlying disorders that put them at increased risk for malignant hyperthermia or inhaled anesthetic-related rhabdomyolysis.
Intraoperative Monitoring	Standard ASA monitors should be applied in all cases. Consideration of the need for invasive monitors, such as an arterial line, should be based on a combination of patient and surgical risk factors.

## Data Availability

No new data were created or analyzed in this study. Data sharing is not applicable to this article.
